# Acupuncture in the Treatment of Abnormal Muscle Tone in Children with Cerebral Palsy: A Meta-Analysis

**DOI:** 10.1155/2023/4662788

**Published:** 2023-03-21

**Authors:** Yan Yuanjie, Xue Jianyi, Xu Jinyan, Huang Mao, Yan Siyang, Yin Zhenjin

**Affiliations:** ^1^The Second Outpatient Department of Hebei University of Chinese Medicine, Hebei University of Chinese Medicine, Shijiazhuang 050000, China; ^2^School of Acupuncture and Message, Hebei University of Chinese Medicine, Shijiazhuang 050000, China; ^3^Department of Child Rehabilitation, The First Affiliated Hospital of Hebei University of Chinese Medicine, Shijiazhuang 050000, China; ^4^School of Basic Medicine, Hebei University of Chinese Medicine, Shijiazhuang 050000, China; ^5^Hebei International Joint Research Center for Dominant Diseases in Chinese Medicine and Acupuncture, Shijiazhuang 050000, China

## Abstract

**Objective:**

To analyse the clinical efficacy of acupuncture and routine treatment in improving dystonia in children with cerebral palsy.

**Method:**

The randomized controlled trials published from the establishment of the databases to August 2022 on acupuncture in the treatment of dystonia in children with cerebral palsy were collected and comprehensively searched in China national knowledge infrastructure (CNKI), weipu (VIP), Wanfang, SinoMed, PubMed, Excerpta medica database (EMBASE), and Cochrane Library. The literature was selected according to the established standards, the quality of the included studies was evaluated, the heterogeneity of the included studies was evaluated with the *I*^2^ test, and the appropriate model was selected for analysis. Sensitivity analysis was used to evaluate the reliability of the results, and a funnel plot was used to evaluate the publication bias.

**Results:**

Fifteen studies were included in the meta-analysis. The control group was treated with routine treatment and acupuncture combined with routine treatment. The outcome index showed that the effect in the treatment group was better: Modified Ashworth Scale score: −0.52, 95% confidence interval (CI) (−0.62 to −0.41), *p* < 0.01. The treatment group showed reduced muscle tension to a greater extent (integral eletromyographic (iEMG) score: standard mean square deviation = −2.97, 95% CI (−4.87 to −1.06), *p* < 0.01). The effective rate in the control group was 74.2% and that in the treatment group was 91.5%, odds ratio = 3.70, 95% CI (2.02–6.78), *p* < 0.01. The funnel plot showed publication bias.

**Conclusion:**

Acupuncture combined with routine training could improve muscle tension abnormalities and improve the efficiency of clinical treatment.

## 1. Introduction

*Chinese guidelines for the rehabilitation of cerebral palsy* (2015) [1] pointed out that paediatric cerebral palsy is a syndrome of nonbrain injury caused by various factors. There are many types of cerebral palsy with various clinical manifestations, among which the change in muscle tone is the most common and main manifestation. The spasmodic type, ataxia type, and mixed type all have symptoms of abnormal muscle tone [2]. Therefore, the improvement of muscle tension is an essential and difficult point in the treatment of paediatric cerebral palsy [3]. Currently, rehabilitation training is the main treatment. Functional training can alleviate the condition to a certain extent, but the overall effect is not ideal. Acupuncture, as a traditional Chinese medicine therapy, currently provides a new idea for the treatment of infantile cerebral palsy [4]. In recent years, many studies have pointed out that acupuncture can improve the abnormal muscle tension of children with cerebral palsy. However, the existing research lacks large samples and high-quality clinical trials. The purpose of this study was to evaluate the clinical efficacy of acupuncture in the treatment of dystonia in children with cerebral palsy through meta-analysis and to provide an evidence-based medical foundation for the clinical treatment of dystonia in children with cerebral palsy.

## 2. Data and Methods

### 2.1. Study Inclusion and Screening Criteria

#### 2.1.1. Study Type

Randomized controlled trials (RCTs) of acupuncture in the treatment of abnormal muscle tone in children with cerebral palsy were included.

#### 2.1.2. Diagnostic Criteria

*Chinese guidelines for the rehabilitation of cerebral palsy* [1]: the study subjects met the definition and diagnostic conditions for paediatric cerebral palsy discussed and adopted at the National Symposium on paediatric cerebral palsy in 2006 [5]; the revision of the definition, classification, and diagnosis of cerebral palsy at the Sixth National Children's rehabilitation conference and the 13th National Academic Conference on the rehabilitation of children with cerebral palsy and international academic exchange conference [6]; and the guidelines for diagnosis and treatment of common diseases in paediatrics for traditional Chinese medicine [7]. The specific neurological components [8] include: (1) children with cerebral palsy caused by nonprogressive brain injury; (2) central dyskinesia or growth retardation; (3) postural disorders and abnormal muscle tone; (4) accompanying sensory and perceptual disorders, intellectual disorders, epilepsy, communication disorders, behavioural abnormalities, and other symptoms; and (5) typical symptoms seen in infancy.

#### 2.1.3. Interventions

The control group was treated with routine treatment (excluding acupuncture treatment), and the treatment group was treated with acupuncture combined with routine treatment.

#### 2.1.4. Outcome Indicators

The main outcome measure was the Modified Ashworth Scale (MAS) muscle tone score. The secondary outcome measures were the integral eletromyographic (iEMG) gastrocnemius myoelectric score and effective rate.

#### 2.1.5. Exclusion Criteria

Republished literature, documents for which valid data could not be obtained, and documents with obvious errors in the original text were excluded.

### 2.2. Retrieval Strategy

The China national knowledge infrastructure (CNKI), weipu (VIP), Wanfang, China biology medicine (CBM), PubMed, Excerpta medica database (EMBASE), and Cochrane Library English databases were searched. The retrieval time limit was from the establishment of the database to August 2022. The retrieval adopted the combination of subject words and free words. Key words included cerebral palsy, infantile cerebral palsy, cerebral palsy, acupuncture, muscle tone, muscle tonus, muscle tension, and RCT.

### 2.3. Literature Screening and Data Extraction

Duplicate documents were eliminated using the Note Express software; the titles and abstracts were browsed, and documents were excluded that did not meet the standards or were repeatedly published. We read the full texts further and extracted the data. Two researchers independently completed the literature screening, data extraction, and cross checking. If there was disagreement, a third party intervened and decided after discussion. Data extraction information included the following: (1) basic information about the paper: title, first author, and year of publication; (2) basic information about the research: sample size, diagnostic criteria, intervention measures, course of treatment, and outcome indicators; and (3) literature research methods and quality evaluation: randomization method, blind method implementation, hiding method, and whether the data are complete.

### 2.4. Quality Evaluation of the Included Documents

The included studies were evaluated according to the offset risk assessment criteria recommended by the Cochrane manual. There are six main criteria: random allocation method, allocation concealment, blind setting, data integrity, selective reporting results, and other bias.

### 2.5. Statistical Processing

The RevMan software, version 5.3, was used for the meta-analysis of outcome indicators. The MAS muscle tone score and iEMG gastrocnemius muscle electrical score were continuous variables and are expressed as the standard mean square deviation (SMD) and 95% confidence interval (CI). The effective rate was a binary variable, expressed by the odds ratio and 95% CI. In the chi-square test, if *I*^2^ < 50%, the fixed effect model was used. If *I*^2^ > 50%, it indicated that the heterogeneity of the included study was high. The random effect model was used, sensitivity analysis was performed, and a funnel diagram was used to evaluate publication bias.

## 3. Results

### 3.1. Literature Search

**Figure 1:**
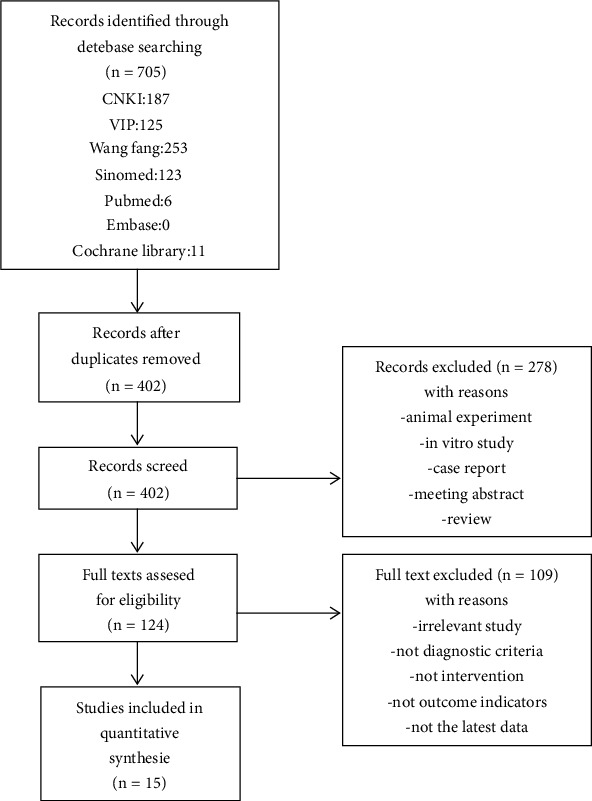
Flowchart of literature screening.

A total of 705 relevant studies were preliminarily searched, and 303 duplicate studies were screened out through the literature manager; titles and abstracts were browsed, 278 were screened out, the full texts were viewed, and the studies that did not meet the inclusion criteria were excluded. Finally, 15 studies were included in the meta-analysis.  Figure 1 shows the literature screening process.

### 3.2. Basic Characteristics and Quality Evaluation of the Included Research

**Figure 2:**
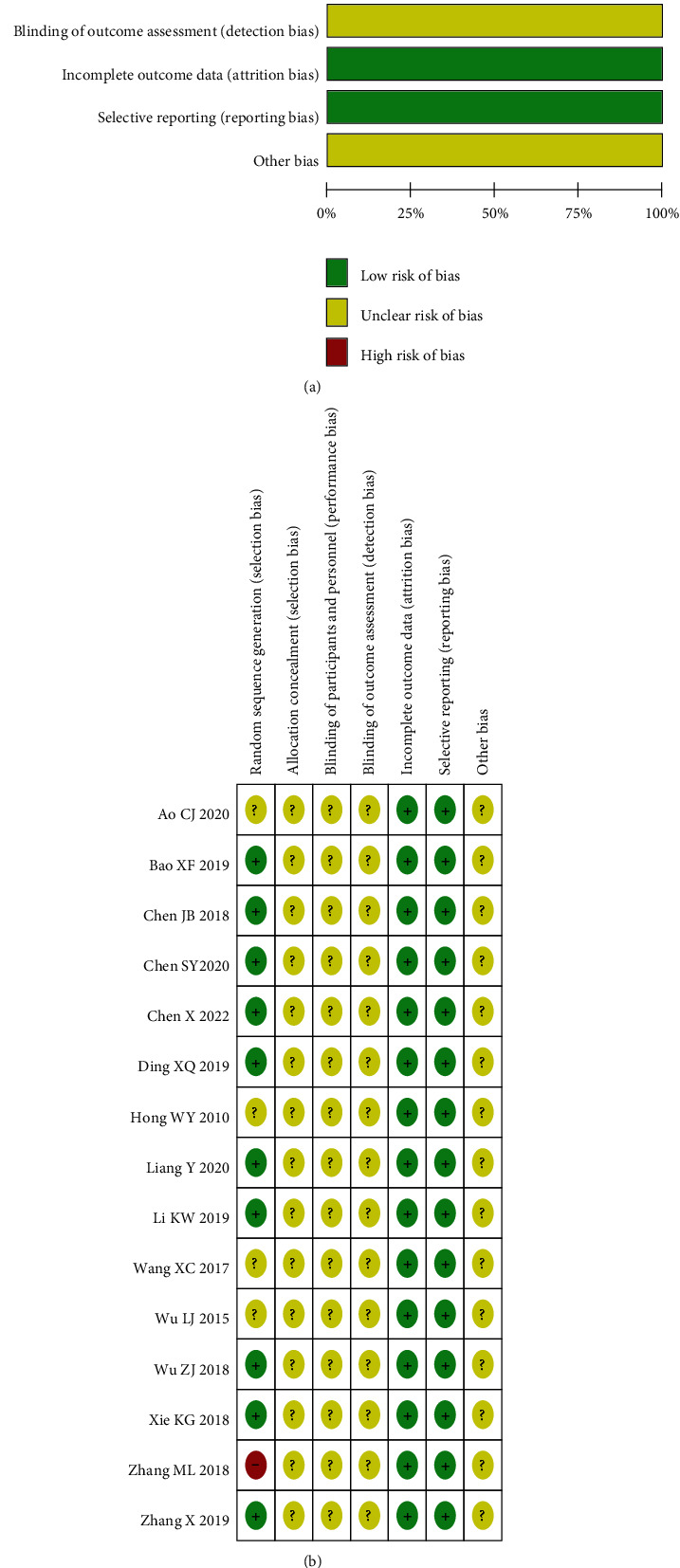
Risk assessment of bias in the included studies.

All of the included studies were RCTs. Among the random allocation methods, 10 were low risk, 9 used the random number table method, and 1 the random lottery method; 4 articles only mentioned randomization, but there was no specific method mentioned, constituting an unknown risk. One article was classified as high risk according to the order of treatment. Allocation concealment and inclusion studies were not proposed, constituting an unknown risk. None of the studies mentioned a blind design, incurring an unknown risk. All data reports were complete, and the measurement and reporting bias was low risk. Other biases were not involved and were unknown risks. The basic characteristics of the included research are shown in Table 1, and the literature quality evaluation is shown in Figure 2.

### 3.3. Meta-Analysis

#### 3.3.1. Comparison of MAS Muscle Tone Scores

**Figure 3:**
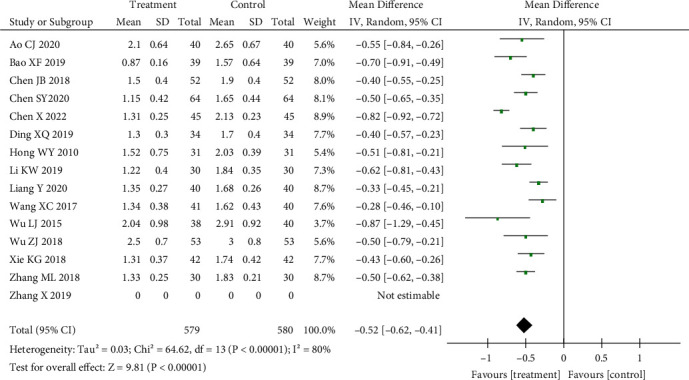
Effect of acupuncture on MAS score.

The MAS muscle tone score was included in 14 studies. First, heterogeneity was tested. There was great heterogeneity (*p* < 0.01, *I*^2^ = 80%). The random effect model was selected: SMD = −0.52, 95% CI (−0.62 to −0.41), *p* < 0.01; and the difference was statistically significant. Acupuncture combined with routine treatment could reduce muscle tone scores. The results are shown in Figure 3.

#### 3.3.2. Comparison of iEMG Gastrocnemius Electrical Scores

**Figure 4:**
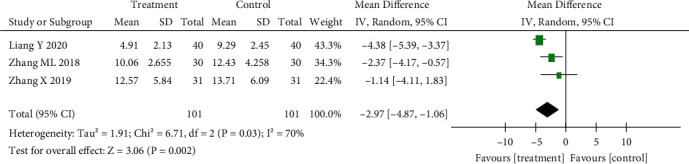
Effect of acupuncture on electrical score of the gastrocnemius muscle.

Three studies were included. The heterogeneity test showed great differences (*p* = 0.03, *I*^2^ = 70%). The random effect model was used for analysis: SMD = −2.97, 95% CI (−4.87 to −1.06), *p* < 0.01. The acupuncture combined treatment group had a reduced gastrocnemius myoelectric score. The results are shown in Figure 4.

#### 3.3.3. Efficiency Analysis

**Figure 5:**
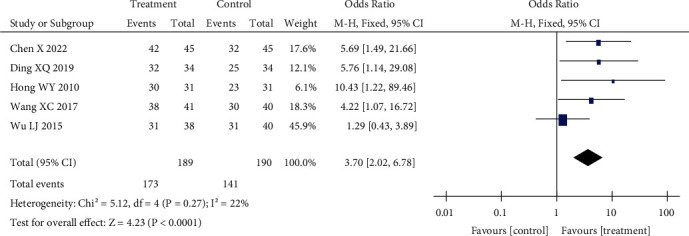
Effect of acupuncture on the effective rate.

Five studies were included in the efficiency analysis. The heterogeneity test showed that the included studies were homogeneous (*p* = 0.27, *I*^2^ = 22%). Using the fixed effect model analysis, the effective rate of the control group was 74.2% and that of the treatment group was 91.5%. The effective rate was 3.70, 95% CI (2.02–6.78), *p* < 0.01), with a significant difference. The effective rate of acupuncture combined with treatment was higher than that of routine treatment. The results are shown in Figure 5.

#### 3.3.4. Sensitivity Analysis

**Figure 6:**
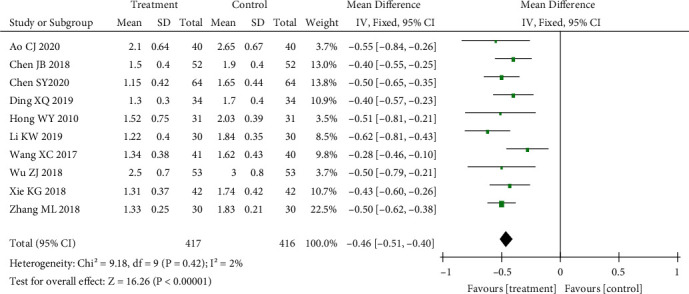
Sensitivity analysis of acupuncture regarding MAS.

*(1) Sensitivity Analysis of the MAS Muscle Tone Score*. After analysing the 14 included studies, it was found that Bao et al. [10], Chen et al. [13], Liang [17], and Wu and Ma [19] could have a great impact on heterogeneity because they were different from other studies in converting the degree of spasm into a score. After removing these four studies, heterogeneity testing was conducted on the remaining 10 studies. The results showed that the heterogeneity was small (*p* = 0.42, *I*^2^ = 2%). After exclusion, the fixed effect model was selected: mean difference (MD) = −0.46, 95% CI (−0.51 to −0.40). The combined effect amount was *Z* = 16.26, *p* < 0.01, and the difference was statistically significant, suggesting that acupuncture combined with treatment could reduce the muscle tone score, consistent with the results before exclusion, and the sensitivity was low. Therefore, the results of the meta-analysis were reliable, and the results are shown in Figure 6.

**Figure 7:**
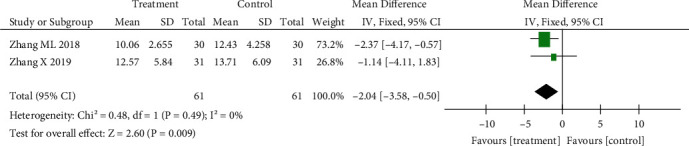
Sensitivity analysis of acupuncture regarding gastrocnemius myoelectric score.

*(2) Sensitivity Analysis of the iEMG Gastrocnemius Electrical Score*. After analysing the three articles included, it was found that Liang [17] measured the electromyography (EMG) score in the passive activity state of children, which was different from the other two studies and could have a great impact on the heterogeneity. After removing the literature, the heterogeneity test showed that there was no heterogeneity (*p* = 0.49, *I*^2^ = 0%), and the combined effect showed *Z* = 2.60, *p* < 0.01, suggesting that acupuncture combined treatment could reduce the EMG score of the gastrocnemius muscle, consistent with the results before exclusion, and the sensitivity was low. The results of meta-analysis were reliable, and the results are shown in Figure 7.

#### 3.3.5. Publication Bias

**Figure 8:**
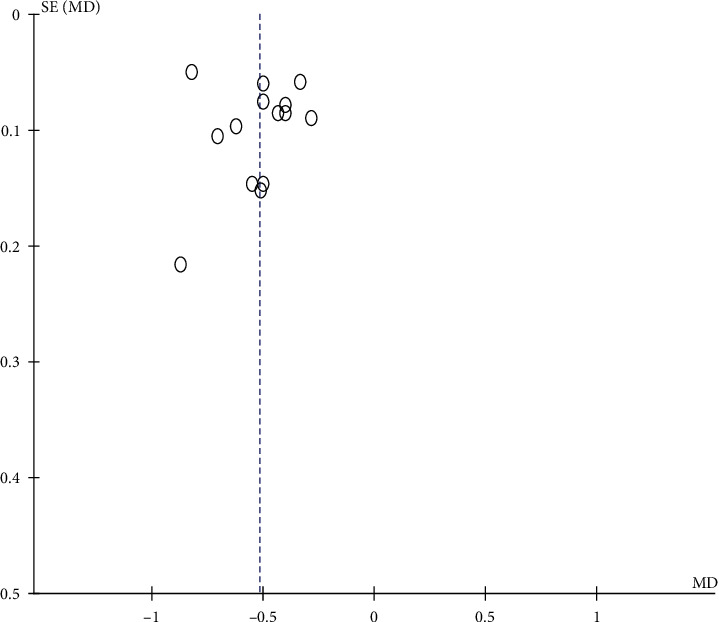
Publication bias of acupuncture regarding improving the MAS score.

The publication bias of acupuncture treatment regarding the MAS score of children with cerebral palsy is shown using a funnel diagram. The distribution of scattered points in the three studies was not completely symmetrical, suggesting that there might be publication bias, which could be related to the heterogeneity between studies. The results are shown in Figure 8.

## 4. Discussion

### 4.1. Mechanism of Acupuncture in the Treatment of Infantile Cerebral Palsy

Traditional Chinese medicine believes that cerebral palsy belongs to the categories of “five lateness,” “five softness,” and “flaccidity.” It mostly arises from congenital deficiency of kidney Qi and acquired loss of the spleen and stomach, as well as congenital foetal deficiency, loss of five internal organs, deficiency of Qi and blood, insufficiency of marrow and sea, and weakness of muscles. The day after tomorrow, Qi and blood are become passive, and muscles and bones are lost in nourishment [24]. All the studies were performed with the method of flat-reinforcing and flat-reducing, and the acupuncture location was the same as that of the control group. The studies by Ao and others [9, 12, 14–19, 23] adopted the combined method of scalp and body acupuncture, and the needle retention time was 30 minutes. There are two researchers of scalp acupuncture method. Chen et al. [11] kept the needle for 60 minutes, and Wu et al. [20] kept the needle for 30 minutes. Bao et al. [10] used the meridian flow method to inject nail, and the needle retention time was 30 minutes. Chen et al. [13] used Jin's three-needle acupuncture, and the needle retention time was 30 minutes. Two researchers used acupuncture along the meridian. The retention time of Xie and Zhu [21] was 30 minutes and that of Zhang and Liu [22] was 15 minutes. The above studies show that acupuncture has a certain therapeutic effect on children with cerebral palsy. As a simple and effective method, acupuncture has the function of dredging the meridians, harmonizing Yin and Yang, and Qi and blood, supporting the body's healthy Qi and eliminating evil Qi. Selecting acupoints based on syndrome differentiation according to the clinical manifestations of infantile cerebral palsy can improve the therapeutic effect. Modern research has shown that children with cerebral palsy have slow sensory nerve conduction velocity and demyelinating changes of the spinal nerve, resulting in obstacles to muscle innervation [25]. After acupuncture treatment, the cerebral blood flow speed of children with cerebral palsy is accelerated, vascular resistance is reduced, Qi and blood are normal, the vein is accessible, and children's functions are restored [26]. Animal experiments have proven that acupuncture can regulate the central bioelectric activity of the cerebral cortex, improve the neural function of cerebral palsy rats, reduce the apoptosis of nerve cells, inhibit the inflammatory response, reduce immune injury, relieve muscle tension, increase the local oxygen supply and blood flow to tissues, and improve the metabolic environment of nerve cells to effectively promote the regeneration of these cells and the improvement of limb function [27–29].

### 4.2. Clinical Effect of Acupuncture on Abnormal Muscle Tone in Children with Cerebral Palsy

Normal muscle tone is very important for maintaining normal posture and promoting movement. In children with cerebral palsy, especially spastic cerebral palsy, dystonia is the main feature. The spasms caused by abnormal muscle tone have a serious adverse impact on children's posture development and motor development [30]. The degree of disability of children with cerebral palsy is also closely related to the severity of spasms [31]. The MAS was published in 1987 [32] and is now widely used at home and abroad. It is one of the main methods used to evaluate the degree of spasm and muscle tension [33]. Therefore, it was used as the main outcome index to reflect the degree of muscle tension. Surface electromyography (sEMG) can obtain relevant muscle function through data analysis. The level of the sEMG signal can reflect muscle tension. An increase in the signal indicates muscle tension, and a decrease in the signal indicates muscle relaxation [34]. iEMG is one of the most commonly used time-domain analysis indices of sEMG, and it can reflect changes in muscle tension [35]. This study used the methods of evidence-based medicine to conduct meta-analysis on the collected clinical RCTs of acupuncture to improve the abnormal muscle tension of children with cerebral palsy. The results showed that acupuncture could improve the abnormal muscle tension of children with cerebral palsy and reduce the degree of limb spasm, with advantages over conventional exercise and other methods. In terms of efficiency, the analysis results showed that acupuncture can improve the efficiency of the treatment of children with cerebral palsy. The results showed that acupuncture can improve the abnormal muscle tension of children with cerebral palsy and reduce the degree of limb spasm, demonstrating advantages over conventional rehabilitation treatment. In terms of the effective rate, the analysis results showed that acupuncture can improve the effective rate in children with cerebral palsy. The study found that acupuncture could provide favourable conditions for routine rehabilitation training, provide guarantees for correcting abnormal posture and normal exercise training, and improve abnormal muscle tension [36]. Acupuncture activates the balance regulation of the central nervous system through its unique mechanism and improves the balance ability of the body and the efficiency of rehabilitation training. In each study, there was no significant difference in the gender of children with cerebral palsy, and the baseline level was the same, so the gender had no significant impact on the overall treatment. When studying whether the acupuncture time affects the overall sensitivity, because the treatment time of three studies [11, 12, 22] is different from that of other studies in 30 minutes, the acupuncture time of two studies [11, 12] is prolonged, and the acupuncture time of Zhang and Liu [22] is shortened to 15 minutes. After removing these three studies, the sensitivity is inconvenient and has no impact on the overall sensitivity. When studying whether age affects the overall sensitivity, because Chen and other researchers [12, 13, 15, 16, 19, 22] will control the age of children with cerebral palsy to be under 6 years old, the six studies will be analysed separately, and the results show that there is no significant impact on the overall sensitivity, so we cannot draw the conclusion that early use of acupuncture therapy will have a better prognosis. In addition, as a green physical therapy, acupuncture has certain advantages regarding convenience, safety, and economy. Therefore, acupuncture can be added to the treatment of cerebral palsy to improve the curative effect of clinical treatment.

### 4.3. Problems and Prospects

At the same time, this study also had some limitations. First, only 15 Chinese studies were included in the meta-analysis, and there were no foreign studies. Therefore, the differences in implementation at home and abroad cannot be evaluated more comprehensively. Second, the quality of Chinese jurisprudence included in the research was low. The overall sample size of each study was small, and there were no large sample or multicentre studies. The random assignment methods in some studies were not introduced in detail. None of the studies involved allocation concealment or a blinded design. There was measurement and implementation bias, which could exaggerate the clinical treatment effect. Third, withdrawal and abscission cases were not mentioned in each study. In addition, none of the studies involved follow-up, and the long-term efficacy could not be observed. Fourth, acupuncture treatment is mostly empirical treatment. There is no unified standard in acupuncture point selection, time, intensity, or manipulation, which might have resulted in heterogeneity in the results. The above deficiencies could have a certain impact on the evaluation results, limiting the demonstration strength of this study to a certain extent.

This study has the following implications for future clinical trials: First, we should strengthen the standardization of clinical diagnostic criteria, inclusion criteria, and efficacy criteria. Second, research designs should be more reasonable and scientific, and high-quality randomized, controlled clinical trials with multiple centres and large samples should be performed, paying attention to the description of randomization methods, allocation concealment, blinding methods, losses to follow-up, adverse reactions, etc. Third, the selection of acupuncture points, time, depth, intensity, manipulation, course of treatment, and other aspects must be deeply studied to render the treatment more scientific and standardized.

In conclusion, acupuncture treatment could improve dystonia in children with cerebral palsy, improve the curative effect of clinical treatment, and showed clinical popularization value in the treatment of dystonia. At the same time, we also hope that the research design and operation will become more scientific and standardized and that the quality of the literature will improve and provide more reliable evidence for clinical acupuncture in the treatment of infantile cerebral palsy.

## Figures and Tables

**Table 1:** Basic characteristics of the included studies.

Inclusion study	Random method	Age	Baseline level	Sample size		Control	Treatment	Outcome	Course of treatment
				Control	Treatment				
Ao et al. [9]	Unknown	3–9 years	Same	40	40	A	AB	①	6 months
Bao et al. [10]	Random number table	6–14 years	Same	39	39	A	AB	①	2 months
Chen et al. [11]	Random number table	0.6–8 years	Same	52	52	A	AB	①	3 months
Chen et al. [12]	Random number table	1–5 years	Same	64	64	A	AB	①	3 months
Chen et al. [13]	Random number table	0.6–6 years	Same	45	45	A	AB	①③	1 month
Ding et al. [14]	Random number table	0.5–7 years	Same	34	34	A	AB	①③	12 weeks
Hong et al. [15]	Unknown	0.3–3 years	Same	31	31	A	AB	①③	1 month
Li [16]	Random number table	2–6 years	Same	30	30	A	AB	①	12 weeks
Liang [17]	Random number table	2–10 years	Same	40	40	A	AB	①②	8 weeks
Wang [18]	Unknown	2–12 years	Same	40	41	A	AB	①③	3 months
Wu [19]	Unknown	0–3 years	Same	40	38	A	AB	①③	3 months
Wu [20]	Random number table	1–10 years	Same	53	53	A	AB	①	12 weeks
Xie [21]	Random draw lots	2–10 years	Same	42	42	A	AB	①	3 months
Zhang and Liu [22]	Visit order	2–6 years	Same	30	30	A	AB	①②	20 days
Zhang et al. [23]	Random number table	0–3 years	Same	31	31	A	AB	②	45 days

Note: A, routine treatment (excluding acupuncture treatment), and B, acupuncture combined with routine treatment. ① MAS muscle tone score, ② iEMG gastrocnemius myoelectric score, and ③ effective rate.

## Data Availability

The CNKI, VIP, Wanfang, CBM, PubMed, EMBASE, and Cochrane Library English databases were searched. The retrieval time limit was from the establishment of the database to August 2022.
